# What Will Ensure a High Standard of Medical Knowledge?

**Published:** 1867-10

**Authors:** 


### WHAT WILL ENSURE A HIGH STANDARD OF MEDICAL KNOWLEDGE?

Thorough qualification exhibited in the green-room, and not additional requisites for admission to examination for the Degree, insures a high standard of medical knowledge in the profession. If Colleges from interested motives, or other cause, fail to make the necessary exactions, a separation of the teaching and licensing authority, by the appointment of Boards for examination unconnected with medical Schools, is the only plan of effecting that desirable object.

The following opinion of the attempt at reform in this particular by the Convention of Colleges, we copy from the *Medical Gazette*, a weekly Review of Practical Medicine, Surgery and Obstetrics, New York; the first No. of which is on our table:

"The reformers of medical schools appear to meet with small success. That reforms are needed, and that the requirements for admission and for graduation should be more severe than at present, is undoubted. We fail, however, to perceive the superior advantages of the schedule proposed by the committee whose report is before us. Anatomy, for instance, is only required to be studied by the freshman class, instead of every session, as it should be. Public hygiene is included in the proposed curriculum, but personal hygiene, which we think of equal if not greater importance, is omitted. Reformers have a hard time of it in this world and we anticipate a rugged path for the estimable and distinguished gentlemen who have the matter in hand."

